# A Systematic Review and Meta-Analysis on the Prevalence of Variants in the Pancreaticobiliary Duct Junction and Its Association with Cancer

**DOI:** 10.3390/biomedicines13051039

**Published:** 2025-04-25

**Authors:** Juan José Valenzuela-Fuenzalida, Antonia Beas-Gambi, Josefa Matta-Leiva, Daniela Martínez-Hernández, Daniel Milos, Mathias Orellana-Donoso, Alejandra Suazo Santibáñez, Alejandro Bruna-Mejias, Andres Sebastian Riveros, Alvaro Becerra-Farfan, Juan Sanchis-Gimeno, Héctor Gutierrez-Espinoza, Carlos Bastidas-Caldes

**Affiliations:** 1 Departamento de Morfología, Facultad de Medicina, Universidad Andrés Bello, Santiago 8370321, Chile; juan.kine.2015@gmail.com (J.J.V.-F.); aantoobeas@gmail.com (A.B.-G.); maajosefa@gmail.com (J.M.-L.); danielamartinezvh@gmail.com (D.M.-H.); danielmilos@gmail.com (D.M.); alejandro.bruna@unab.cl (A.B.-M.); 2 Escuela de Medicina, Universidad Finis Terrae, Santiago 7501015, Chile; mathor94@gmail.com; 3 Department of Morphology and Function, Faculty of Health Sciences, Universidad de Las Américas, Santiago 8370040, Chile; alej.suazo@gmail.com; 4 Departamento de Ciencias Morfológicas, Facultad de Ciencias, Universidad San Sebastián, Lientur 1457, Concepción 4080871, Chile; 5 Departamento de Ciencias Química y Biológicas, Facultad de Ciencias de la Salud, Universidad Bernardo O’Higgins, Santiago 8370993, Chile; alvaro.becerra@ubo.l; 6 GIAVAL Research Group, Department of Anatomy and Human Embryology, Faculty of Medicine, University of Valencia, 46001 Valencia, Spain; juan.sanchis@uv.es; 7 Faculty of Education, Universidad Autónoma de Chile, Santiago 7500912, Chile; kinehector@gmail.com; 8 Facultad de Ingeniería y Ciencias Aplicadas, Biotecnología, Universidad de las Américas, Quito 170125, Ecuador

**Keywords:** pancreaticobiliary junction (PBJ), pancreaticobiliary maljunction, cancer, pancreaticobiliary junction cancer

## Abstract

**Background/Objectives:** The objective of this study was to describe the anatomical variants of the pancreaticobiliary junction and how its position or structural change could be associated with hepatic, duodenal, and pancreatic clinical complications. **Methods:** We searched MEDLINE, Scopus, Web of Science (WOS), Google Scholar, CINAHL, and EMBASE databases from their inception up to September 2024. **Results:** Two authors independently performed the search, study selection, data extraction, and assessed the methodological quality with an assurance tool for anatomical studies (AQUA). Finally, the pooled prevalence was estimated using a random effects model. A total of 59 studies with a total of 22,752 participants were included in this review. The overall prevalence of the anomalous pancreaticobiliary junction (APBJ) variant was 12% (95% CI = 6% to 18%). The prevalence of cancer associated with variants of APBJ was 29% (95% CI = 23% to 34%). **Conclusions:** In the present anatomical systematic review and meta-analysis, we found that a longer common channel correlated with a higher prevalence of bile duct or gallbladder malignancy, due to the backward flow of bile which occurs as a result of the position and distance of the bile ducts, as well as pancreatic failing. Hence, APBJs are of great interest for gastroduodenal surgeons.

## 1. Introduction

The pancreaticobiliary junction (PBJ) forms when the bile duct and pancreatic duct (PD) join to open into the second (descending) part of the duodenum. The hepatopancreatic ampulla is a structure that derives its name as a smooth muscle sphincter, which functions as a regulator of bile flow in the PBJ [1]. This anatomical structure plays an important role as it surrounds the distal end of the PBJ at the level of the medial wall of the descending portion of the duodenum. It allows the exit of the exocrine pancreatic secretions and bile from the liver to the duodenum. Both ducts form a common channel. However, when the channel is longer than usual, the union of the ducts is located outside the duodenal wall, constituting an anomalous union that is not regulated by the pancreatobiliary ampulla (sphincter of Oddi). This condition is called anomalous pancreaticobiliary junction (APBJ) [2,3,4].

The formation of the pancreaticobiliary duct and ampulla of Vater is closely linked to the formation of the liver, pancreas, and duodenum. The liver primordium develops in the middle of the third gestational week or at the beginning of the fourth gestational week. During this period, the foregut arises from the liver bud or diverticulum inferior to the dilated stomach. Liver bud cells proliferate and advance into the transverse septum (posterior diaphragm). During its advancement, it leaves a duct that connects the liver bud and the foregut, a duct that will form the biliary tree. From this duct a different bud is formed and generates the gallbladder (GB), allowing the ventral pancreatic bud to appear next to this [5,6]. The pancreas begins to form during the fifth gestational week with the aforementioned bud. The dorsal pancreatic bud originates from the foregut. These two structures then fuse together, forming the main PDs joined with the bile duct and the accessory duct if the proximal part of the dorsal pancreatic bud is not obliterated [6,7]. For the formation of these structures, there must be correct and coordinated signaling of their mediators in the different embryonic processes through which these organs pass. Therefore, failure of or defective signaling could potentially cause malformations, such as APBJ. This type of malformation—in which the junction of the bile and PDs occurs prior to their contact with the duodenum—will generate different anatomical variants in the junction, leading to poor function of the sphincter of Oddi that leads to the reflux of pancreatobiliary juices, increased intraductal pressure, and stasis of secretions, making it a predisposing factor for pancreatitis and bile duct cancer [8,9]. APBJ is congenital and can potentially result in two different types of regurgitation. These two types of potential regurgitation are pancreatobiliary and biliopancreatic refluxes. Being a longer common channel, the pancreatobiliary ampulla acts without affecting the union of the pancreaticobiliary ducts. Consequently, a prolonged exposure of the epithelia to certain substances, such as activated pancreatic enzymes and free bile acids, can occur. One of the major side effects of this treatment is chronic inflammation, which favors the proliferation of epithelial cells and hyperplasia. Many other pathologies may occur at the level of the biliary tract and pancreas, such as pancreatitis, bile duct dilation, and malignant neoplasms of the GB and bile ducts, among others [10,11]. The pathogenesis of pancreatobiliary malformation has not been fully elucidated; however, it probably develops because of a malformation of the ventral pancreas when the PD system joins the bile duct at approximately the fourth gestational week [12,13]. The histology of the hepatopancreatic ampulla or ampulla of Vater is similar to that of the GB, except that the former has a tall cuboid rather than a columnar epithelium and can be affected by the reflux of bile and pancreatic secretions. However, studies that more thoroughly explain the histopathological changes in the Vater ampulla are lacking in the current medical literature. Alterations in this structure are rare, and most studies focus on the histological changes in GB pathologies. Muraki et al. [14] showed that the histopathological changes in the GB are detailed with respect to the reflux of bile and pancreatic juices, in which changes in epithelial thickness were found to cause a smaller GB. Defective muscular layers promote the accumulation of bile residues in the PBJ, bile ducts, or PDs.

Various imaging techniques have been used to diagnose this condition. Some of the techniques currently used include direct cholangiography, endoscopic retrograde cholangiopancreatography, resonance cholangiopancreatography, and three-dimensional (3D) drip infusion cholangiography with computed tomography (CT). This last technique allows physicians to detect alterations such as an abnormally long channel, common ducts, and even the presence of an abnormal junction between the bile and PDs. Endoscopic ultrasonography is another technique currently used in modern medicine to diagnose these conditions. This is possible because the PBJ is located outside the duodenal wall. It can be diagnosed by performing an anatomical examination during surgery or autopsy [15,16,17,18].

**Figure 1:**
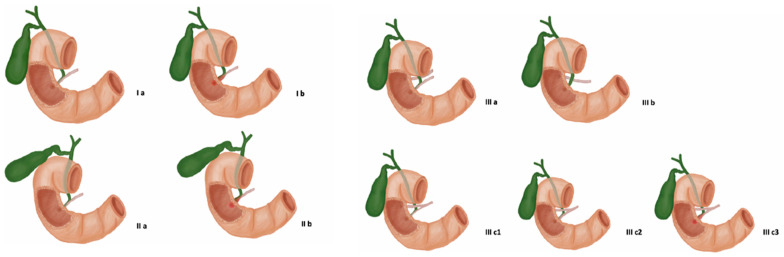
Variants of the pancreaticobiliary junction—a representation of the Komi classification of anomalies of the biliopancreatic junction (APBJ). Type I: the angle of the joint C-P is perpendicular; Ia without dilatation of the common duct; and Ib with dilation of the common duct. Type II: obtuse-angle P-C junction; and IIa without dilatation of the common duct; IIb with dilatation of the common duct. Type III: complex APBJ; IIIa is the complete pancreas divisum with biliary dilatation; IIIb represents an absence of the main pancreatic duct; IIIc1 presents a small communication duct between the main and accessory pancreatic duct; IIIc2 shows that the communication between the main and accessory duct is of the same caliber as the ducts; and IIIc3 is the same as type IIIc2, but with dilated ducts.

Two common methods used to classify the APBJ are the Komi and the Kimura classifications. The classification proposed by Kimura [19] classified these anomalies into two varieties according to the mouth of the PD and the bile duct in the common bile duct (CBD). In the previously mentioned study, the latter was abnormally long, as previously stated. Kimura type I is called P-C or pancreaticobiliary, accounting for only 15–20% of cases. In contrast, Kimura type II’ is called C-P or biliopancreatic and it is the most common form, accounting for 80–85% of cases [19]. The difference is that in type I, the PD empties into the bile duct; however, in type II, the bile duct empties into the PD. A more complex classification approach is proposed by Komi et al. [20] as it considers the involvement of the common duct and also the angles between the bile duct and the PD. This classification presents the following types: types Ia and Ib; types IIa and IIb; and types IIIa, IIIb, IIIc1, IIIc2, and IIIc3 [21], which are detailed in Figure 1.

Bile duct alterations have been studied for several decades and reflect the high prevalence of bile duct cancer. Patients with APBJ have a higher probability of developing cancer [22,23]. Roukounakis [23] showed that among 29 participants, 13 (45%) had APBJ, and notably, all 13 presented with malignancy. The study also reported that 2 individuals (15.4%) developed GB carcinoma, and 11 individuals (84.6%) developed bile duct carcinoma. Moreover, earlier case reports yielded a 100% prevalence of malignancy among patients with APBJ [22]. Therefore, in the presence of these abnormalities, therapeutic or surgical treatment is recommended to prevent serious damage to the bile duct [22,23].

The main objectives of this systematic review and meta-analysis were to describe the prevalence of anatomical variants of the PBJ and to report the prevalence of cancer in participants with such variants. Second, the following specific objectives were examined: (1) to understand the morphology of the APBJ, (2) to identify the type of APBJ, (3) to assess the clinical implications associated with the presence of an APBJ, (4) to determine and describe the prevalence of cancer linked to an APBJ.

## 2. Methods

### 2.1. Protocol and Registration

This systematic review and meta-analysis was performed and reported according to the Preferred Reporting Items for Systematic Reviews and Meta-Analyses (PRISMA) statement [24]. The registration number in the International Prospective Register of Systematic Reviews (PROSPERO) is CRD42022224066.

### 2.2. Eligibility Criteria

Studies on the presence of APBJ variants and their association with any clinical condition were considered eligible for inclusion if the following criteria were met: (1) population: cadavers or dissection specimens, APBJ imaging, or patients with diagnosed APBJ intrasurgically; (2) results: presence of APBJ, presence of APBJ plus clinical complications (hepatic, pancreatic, and duodenal) diagnosed by imaging, cadaveric, and/or surgical means, and symptoms that can be confirmed by a professional or through different exams; (3) studies: inclusion of research articles, research reports, or original research published in English or Spanish in peer-reviewed journals and indexed in the reviewed databases (listed in Section 2.3). On the contrary, the exclusion criteria were the following: (1) population: animal studies; (2) studies that analyzed variants of the ductal system outside the PBJ; and (3) studies published as letters to the editor or comments.

### 2.3. Electronic Search

We systematically searched MEDLINE (via PubMed), Web of Science, Google Scholar, Cumulative Index to Nursing and Allied Health Literature (CINAHL), Scopus, and EMBASE from their inception until September 2024.

The search strategy included a combination of the following terms: “Anomalous pancreaticobiliary junction” (No MeSH), “Mal Junction Pancreaticobiliary” (No MeSH), “Cancer” (MeSH terms), “Pancreaticobiliary junction cancer” (No MeSH), “variation anatomical” (No MeSH), “clinical anatomy” (No MeSH), and “Hepatic duct” (No MeSH) using the Boolean connectors “AND”, “OR”, and “NOT”.

### 2.4. Study Selection

Two authors (J.J.V.-F. and M.O.-D.) independently screened the titles and abstracts of the references retrieved from the search. We obtained the full texts of the references that either author considered potentially relevant. A third reviewer (DM) was included if a consensus could not be reached.

### 2.5. Data Collection Process

Two authors (A.B.-G. and H.G.-E.) independently extracted the data on the outcomes of each study. The following data were extracted from the original reports: (1) authors and year of publication; (2) country; (3) type of study; (4) sample characteristics (sample size, age, distribution, and sex); (5) prevalence and morphological characteristics of APBJ; (6) statistical data reported by each study; and (7) main results.

### 2.6. The Assessment of the Methodological Quality of the Included Studies

A quality assessment of the retrospective and prospective observational studies was performed using the methodological quality assurance for anatomical studies (AQUA) tool proposed by the International Evidence-Based Anatomy Working Group [25]. Two reviewers (J.J.V.-F. and J.M.-L.) independently performed the data extraction and quality assessment. A third reviewer (H.G.-E.) was involved if a consensus was not reached. For case study bias, the authors (D.M. and M.O.-D.) independently assessed the risk of bias in the included studies. To assess the risk of bias in case reports belonging to the descriptive study category, we used the Joanna Briggs Institute critical appraisal checklist for case reports [26]. Each article was evaluated using 8 questions with potential responses of “yes”, “unclear”, “no”, or “not applicable.” Articles were evaluated using the following criteria: (1) low risk of bias: more than 70% of the “yes” score; (2) moderate risk of bias: 50–69% of the “yes” score; (3) high risk of bias: less than 49% “yes” score. Two authors independently applied the tool to each article to arrive at an overall judgment with supporting justifications.

### 2.7. Statistical Methods

The data extracted from the meta-analyses were interpreted by calculating the prevalence of APBJ variants and processing the information using the R statistical software (Version 5.1.12, https://posit.co/download/rstudio-desktop/, accessed 1 October 2024) [27]. The prevalence of an APBJ variant was considered an effect size if it was less than 35%, to distinguish it as a variant rather than variability. The effect size of the relationship between APBJ and cancer was considered significant if it was greater than 25% for this anatomoclinical relationship [27]. The DerSimonian–Laird model with Freeman–Tukey double arcsine transformation was used to combine the summarized data. Furthermore, a random effects model was used because the APBJ prevalence data were heterogeneous. It should be noted that, in order to calculate the prevalence, only studies with more than 20 participants were included, since with smaller numbers the reported data could be overestimated. The degree of heterogeneity among the included studies was assessed using the chi-square test and the heterogeneity statistic (I^2^). For the chi-square test, the *p*-value proposed by the Cochrane Collaboration was considered significant when it was <0.10. The I^2^ statistic values were interpreted with a 95% confidence interval (CI) as follows: 0 to 40% might not be significant, 30 to 60% might indicate moderate heterogeneity, 50 to 90% might represent substantial heterogeneity, and 75 to 100% could represent a significant amount of heterogeneity [25]. Additionally, the odds ratio of cancer development (CDOR) and 95% CI of the studies included in the meta-analysis were recorded, and if the study did not report it, it was calculated following the guidelines described by Karla [28].

## 3. Results

### 3.1. Included Articles

**Figure 2:**
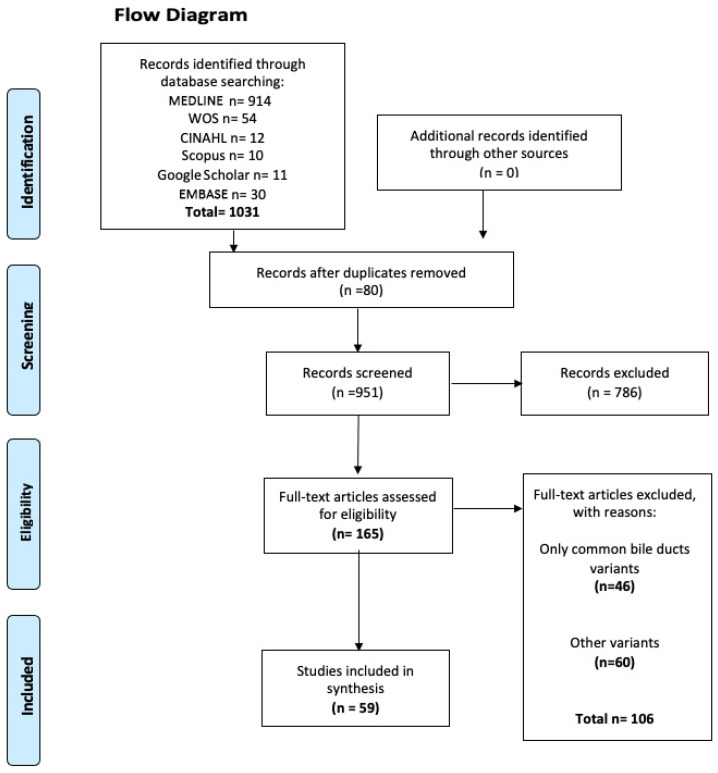
Flow diagram of study selection according to PRISMA.

The search yielded a total of 1031 articles from various databases, all of which met the criteria and search terms established by the research team. The filter was applied to the titles and/or abstracts of the articles in the searched databases, and the primary criterion of elimination of duplicates was used. In total, 165 full-text articles were evaluated for eligibility for inclusion in this meta-analysis and systematic review. Next, 111 studies were excluded because their primary and secondary results did not match those of this review or because they did not meet the established criteria for good data extraction, resulting in 59 articles [2,3,4,5,9,12,13,14,15,20,23,29,30,31,32,33,34,35,36,37,38,39,40,41,42,43,44,45,46,47,48,49,50,51,52,53,54,55,56,57,58,59,60,61,62,63,64,65,66,67,68,69,70,71,72,73,74,75,76] (Figure 2) being included for analysis (n = 22,723 patients, images, and cadavers) (Supplementary Table S1).

#### 3.1.1. The Geographical Characteristics of the Studies and Participants

**Figure 3:**
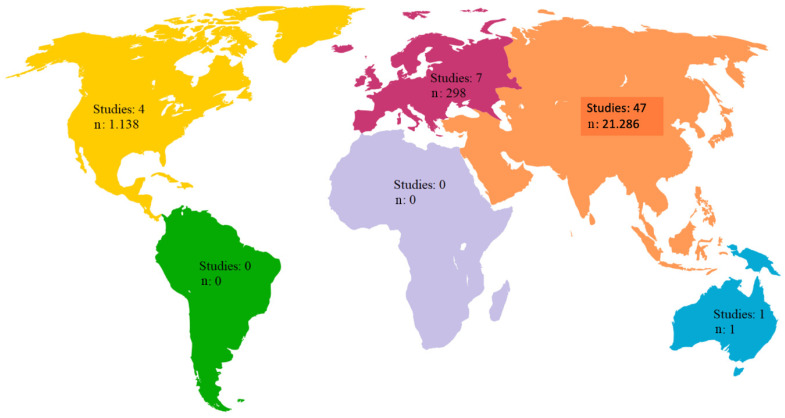
Geographic distribution of reviewed studies and total number of participants included in studies.

This review included a total of 59 studies conducted in various continents, with the exception of Africa and South America (Figure 3). The distribution of studies across continents is as follows:

**Europe**: Seven studies, representing 11.86% of the included studies, with a cumulative number of 298 patients, equivalent to 1.31% of the total samples reviewed.

**Asia**: Forty-seven studies, equivalent to 79.66% of the included studies, with a cumulative number of 21,286 patients, representing 93.67% of the samples included in the analysis.

**North America**: Four studies, equivalent to 6.77% of the included studies, with a cumulative number of 1138 patients, representing 5.01% of the total sample size included in the analysis.

**Oceania**: One study, accounting for 1.69% of the included studies, with a cumulative number of one patient, equivalent to 0.004% of the samples analyzed.

#### 3.1.2. Sex and Age Characteristics in Studies with Sample Size Greater than One

For the 38 studies with a sample size greater than one, the participant data that were collected included sex (Supplementary Table S1; Table 1) and average age (Supplementary Table S1).

- **Sex of Participants**:

Six studies did not report the sex of participants [4,5,13,31,32,74], representing a total of 3707 participants, which is equivalent to 16.31% of the total samples of studies with a sample size greater than 1. Three studies had only female participants (n = 11) in their analysis [43,44,70], which was equivalent to 0.05% of the total sample of studies with a sample size greater than 1. In total, 38 studies included both male and female participants in their sample, with the total number of male and female participants being 537 (2.36%) and 1072 (4.71%) of the total sample of studies with a sample size greater than 1, respectively, and for a total of 17,227 (75.81%) patients of these 38 studies, sex was not reported. The percentage of male subjects ranged from 0.15% to 51.61%, with an average of 19.03%, and the percentage of female subjects varied between 0.06% and 91.66%, with an average of 37.74%. Finally, the average age reported among all the included studies was [42.41] with an age range between 0 and 90.

#### 3.1.3. Variant Classification in Studies

Among the recovered variants, a detailed classification of studies was carried out based on the Komi and Kimura classifications.

**Figure 4:**
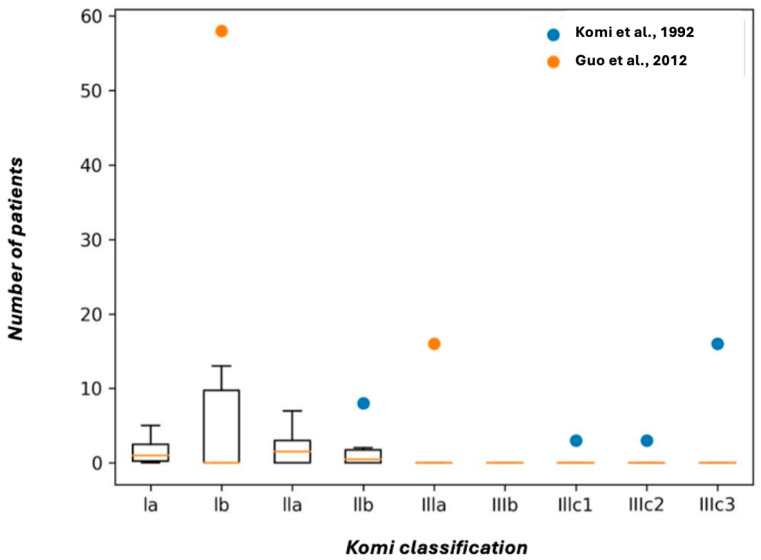
Box plot of studies included in Komi classification [20,53].

**Komi Classification**: Seven studies referred to the Komi classification in the article, with the variants found being more related to type Ia and Ib. A total of 143 variants were cataloged, including 10 cases of type Ia, 71 of type Ib, 13 of type IIa, 11 of type IIb, 16 of type IIIa, 3 of type IIIc1, 3 of type IIIc2, 3 of type IIIc2, and 16 of type IIIc3. No type IIIb variants were found, and in one case, the type could not be determined (Figure 4).

**Figure 5:**
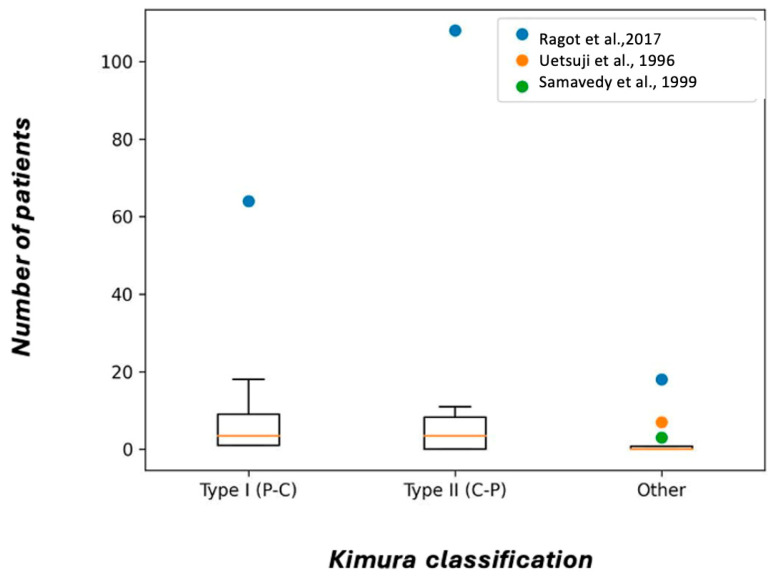
Box plot of studies included in Kimura classification [32,35,75].

**Kimura Classification**: Twelve studies used the Kimura classification, with a total of 302 variants cataloged, including 117 of type I (P-C), 151 of type II (C-P), and 25 with another classification. In three cases, the type was not classified (Figure 5).

### 3.2. Prevalence and Risk of Bias

**Figure 6:**
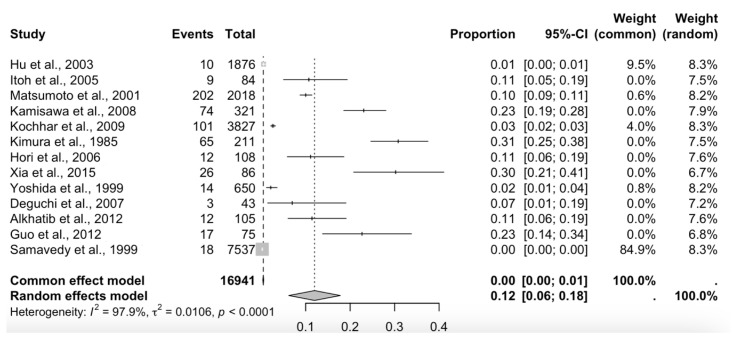
A forest plot of the studies included in the APBJ prevalence analysis [2,3,12,13,15,29,31,34,39,41,42,53,75].

**Figure 7:**
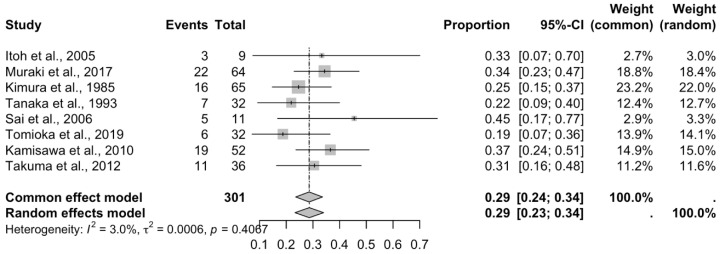
A forest plot of the correlation between APBJ and cancer prevalence [3,14,29,30,71,72,73,74].

**Figure 8:**
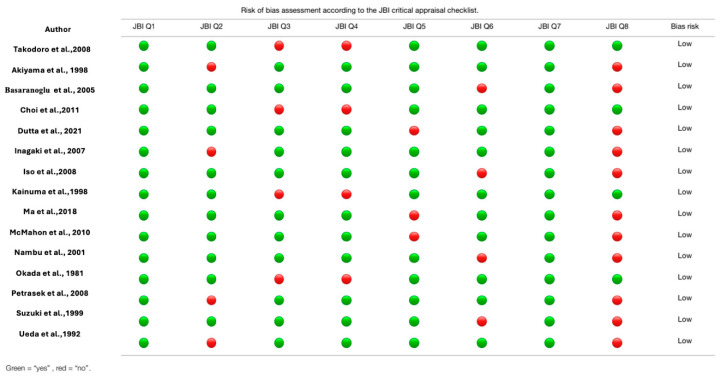
Risk of bias evaluation for case studies [9,56,57,58,59,60,61,62,63,64,65,66,67,68,69].

**Figure 9:**
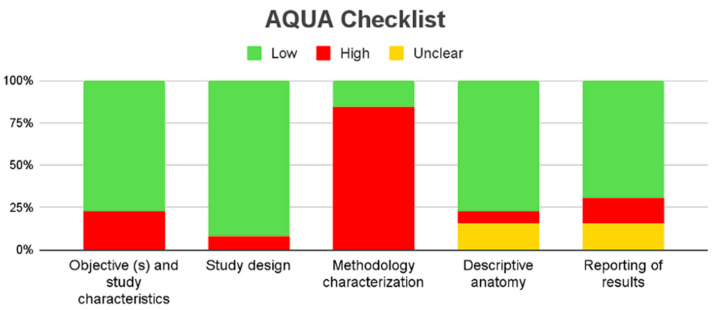
Assessing risk of bias in observational studies [3,6,8,16,17,18,19,20,21,22,23,24,25,26,27,28,29,30,31,32,33,34,35,36,38,39,41,45,57].

Thirteen studies reporting the prevalence of APBJ were included; these met the criterion of presenting a number less than 35% of subjects with the variant described above [2,3,12,13,15,29,31,34,39,41,42,53,75], with a prevalence of 12% and a confidence interval of 6 to 18%, showing a heterogeneity of (I^2^ = 97.9%) (Figure 6; Table 2). Eight studies were included for this meta-analysis of the prevalence of cancer in patients with APBJ [3,14,29,30,71,72,73,74], with a prevalence of 29% and a CI of 23–34%, showing a heterogeneity of (I^2^ = 3%) (Figure 7; Table 3). Regarding the risk of bias in case studies, 100% of the articles analyzed presented a low risk of bias. When we analyzed the different items of the risk of bias for case studies, only question 8 “Does the case report provide lessons for implementation?” presented a high risk of bias in 11 of the 15 articles analyzed [9,59,60,61,62,63,64,65,66,67,68,69,70,71,72] (Figure 8). For the bias analysis using AQUA, 29 studies were included [3,6,8,16,17,18,19,20,21,22,23,24,25,26,27,28,29,30,31,32,33,34,35,36,38,39,41,45,57], of which the main bias presented by the studies was the methodological characterization of 22 of the 29 studies, and the other items are reported in the AQUA checklist presented under the risk of bias (Figure 9).

With respect to the odds ratio, detailed in Table 4, and the data predictor factor associated with the CDOR and the 95% CI as well as the *p*-value, from the eight studies [3,14,29,30,71,72,73,74], only Takuma et al. [74] found that a bile duct diameter greater than 10 mm had a CDOR (95% CI) of 0.67 (0.29–1.58) and *p* = 0.366 in participants who had amylase levels greater than 10,000 IU/L. Furthermore, those participants who had a common canal length greater than 5 mm had a CDOR (95% CI) of 5.57 (2.13–14.5), *p* < 0.001, and amylase levels greater than 10,000 IU/L. For the remaining studies, CDOR and 95% CI were calculated as described by Kalra [28]. In some studies [3,14,29,30,71], a correlation was found between the common channel’s length and malignancy. Reference [71] found that a common channel length greater than 5.3 mm had a CDOR (95% CI) of 42 (501.61–3.51), whereas Kamisawa [73] found that for a common channel length greater than 6 mm, the CDOR was 0.12 (5.42–0.002). In the study by Muraki [14], it was found that in patients with high amylase levels (>10,000 IU/L), the CDOR was (95% CI) 54 (6666.025–0.43), whereas for Itoh [3], the CDOR (95% CI) was 0.007 (0.16–0.0003) for a common channel length > 15 mm. In Tanaka’s study [30], the CDOR (95% CI) was 0.07 (2.62–0.0002), and in Kimura’s study [29], the CDOR (95% CI) was 1.4 (3–0.67). In contrast, Tomoika [72] reported a CDOR (95% CI) of 0.012 (0.21–0.0007) with *p* = 0.005 in participants who had high levels of interleukin (IL)-6 and IL-33, while Takuma [74] reported a CDOR (95% CI) of 3.21 (169.56–0.06) in participants with histopathological findings such as hyperplastic changes (88%), as well as in those with the presence of hypertrophic muscular layers (63%), subserosa fibrosis (88%), adenomyomatosis (63%), gallstone (25%), Ki-67 labeling index (6%), and K-ras mutation (50%) (Table 4).

### 3.3. Clinical Implications

Regarding the clinical issues reported in this review, five studies did not report any associations between clinical findings and APBJ [5,47,56,63,64]. On the other hand, 45 studies reported clinical findings related to APBJ, of which 29 studies reported an association between APBJ and neoplasms of the GB, bile ducts and/or pancreas [2,4,12,13,36,38,39,40,41,45,46,49,50,56,58,59,60,61,62,65,66,69,71,73,77,78,79,80,81]. One study reported that GB and bile duct cancers are not associated with the APBJ [31]. One study reported that APBJ is associated with biliary stenosis [82], defined as a narrowing of the bile ducts which presents different etiologies [83]. Thirteen studies suggested that bile duct cysts are associated with APBJ [32,33,34,35,36,37,38,39,40,41,42,43,44,45,46,47,48,49,50,51,54,58,60,61,73,75,77,81,82,84]. These cysts are congenital malformations which are defined as a dilatation of the bile ducts, presenting more frequently in females and in individuals of Asian origin [85,86]. Thirteen studies reported the association of APBJ with chronic pancreatitis [15,20,34,35,36,37,38,39,40,41,42,43,44,45,46,47,48,49,50,51,52,53,57,60,67,72,73,83,84,87], which refers to the inflammation of the pancreas due to the progressive and irreversible destruction of its cells with subsequent replacement of these by fibrous tissue, which determines the loss of pancreatic function and atrophy of the organ [88]. Five studies showed an association between the appearance of stones in the bile duct and APBJ [20,45,52,70,82], which is defined as the formation of stones in the GB or bile ducts that can lead to complications such as cholecystitis, choledocholithiasis, cholangitis, and acute pancreatitis [89]. It should be noted that in one of these studies, the presence of gallstones was reported in all cases of APBJ type IIIc as per the Komi classification [20], which could be a predisposing factor for choledocholithiasis and other alterations in the structure of the GB bile duct [70]. In addition, in two studies [64,90], APBJ was related to benign tumors; in one of them, it was correlated with vesicular adenomyomatosis generated by the chronic stimulation of the mucosa by the reflux of pancreatic juice in one case with APBJ type P-C [65]. This corresponds to the invagination of the mucosa with the consequent formation of Rokitansky–Aschoff sinuses accompanied by smooth muscle hyperplasia, which may contain thick bile, mucus, or calculi that generate inflammatory, fibrotic, and metaplastic changes [90]. Hyperplasia and proliferative activity of the vesicular mucosa have also been described, in which pancreatic juice reflux is a possible etiological factor [44,65]. In one study, APBJ was related to biliary atresia, which was present in all cases of APBJ type P-C [44]. This condition is described as an obstructive cholangiopathy of unknown etiology that affects the intrahepatic and extrahepatic bile ducts and is present mainly in newborns [76]. One study showed an association between APBJ and liver fibrosis [49], which corresponds to the deposition of extracellular matrix in the liver parenchyma that can progress to cirrhosis [91]. One study reported that APBJ is associated with gastroesophageal reflux with pancreatic contents [68]. Gastroesophageal reflux occurs when a failure in the anti-reflux barrier results in the abnormal passage of digestive secretions into the esophagus, thereby damaging it [92,93]. Another study associated APBJ with altered bile composition [52], which may be secondary to bile stasis and the reflux of pancreatic juice into the bile duct that occurs in APBJ, which are particularly relevant in that they have implications for the development of neoplasms in the biliary tract [52]. Lastly, for the prevalence of cancer associated with APBJ, eight studies showing a prevalence of less than 100% were included (Table 3), in which it turned out that 19% of the patients presented cancer, with an SD of 13–26.

## 4. Discussion

This systematic review and meta-analysis aimed to report the prevalence of APBJ variants and their association with pathologies of the GB, bile ducts, PDs, and hepatopancreatic ampulla. The main finding of our review was the correlation between the prevalence of cancer and its development in the presence of APBJ. Although this correlation was not statistically significant in the included studies, it is a condition to be kept in mind by treating physicians in the region.

We highlight that, based on our search, seven years have passed since the last anatomical review of the APBJ, which was that of Kamisawa in 2017 [18]. The reviews by Kamisawa [18,93] showed the relationships between the variant and its clinical conditions but did not address in detail the presence of cancer or the pathomechanical relationships that cause it. Our study addresses these aspects. In the reviews by Ono et al. [94,95] the researchers found a relationship between APBJ and clinical conditions, but they did not directly address the relationship with cancer, focusing instead on other conditions such as pancreatitis. Furthermore, these reviews did not detail the anatomical features of APBJ.

Considering the factors that could influence the relationship studied in this review, based on sex, 30 studies included both men and women. Although this number was low compared to the total number of included studies, these studies did not report a difference between men and women; therefore, no association was observed between the variants and the sex of the patients. Furthermore, in the context of geographical distribution, the majority of the included studies were carried out in Asia; therefore, it represents the largest number of samples analyzed. This is a limitation, although we conducted an expanded literature search; reports from other continents were missing, so a more homogeneous geographical distribution was not possible. Consequently, we could not infer whether APBJ is influenced by ethnic factors. For the anatomical description characteristics of APBJ, according to the Komi classification, 8 studies reported this classification as Ib; while for the Kimura classification, 12 studies were included, and the most reported types were type I and type II. Importantly, although some studies reported these classifications, clinical studies in particular did not report them. Therefore, we infer that the classification systems remain an anatomical description not used by clinical professionals. Clinical professionals preferably report disposition and trajectory in relation to the anatomical planes, which may indicate a lack of unified language but this is not understood as a clinical limitation, rather an aspect of descriptive anatomy.

Concerning the prevalence of APBJ, the studies that met the inclusion criteria reported a prevalence of 9%. The literature shows that the prevalence is between 1.5 and 3.2% [41]. We may have overestimated the prevalence because the included studies specifically selected patients with APBJ; however, this was not reported in the analyzed articles. In the context of cancer prevalence in patients and cadaveric samples with APBJ, 11 studies were included in the meta-analysis, showing a prevalence of 21%, indicating that one in five patients could suffer from GB cancer or bile duct cancer. Regarding the calculation and interpretation of the effect size based on the information provided, the effect size between different studies is calculated using the prevalence of the variant and the association between its presence and the appearance of cancer in certain patients. It is worth noting that the presence of cancer was associated with variants in some participants. Regarding the APBJ comparison, the effect size of this comparison, as reported, is 0.319, which is not considered statistically significant according to all the studies included and analyzed. It is important to consider the context and specific details of the studies involved when interpreting effect size and statistical significance. Different studies may have different methodologies, sample sizes, and other factors that may influence these calculations. Moreover, we assessed the cancer development odds ratio (CDOR) of the included studies in the meta-analysis to determine the relationship between APBJ (exposure) and cancer presentation (outcome). These data, detailed in Table 4, show that only the study by Horaguchi et al. [77] reported the CDOR and 95% CI with *p* > 0.001 for a common channel length > 5 mm, as well as the presence of acute pancreatitis. This may be due to a loss of functionality of the sphincter of the major duodenal papilla, resulting in repeated involvement of the biliary epithelium, activating oncogenes, and inactivating tumor suppressor genes, inducing the hyperplasia–dysplasia–carcinoma sequence. The reflux of pancreatic juice towards the bile duct and the reflux of bile towards the PD lead to a bidirectional flow and stagnation of the mixture of bile and pancreatic juice, which in turn leads to the development of acute pancreatitis. The CDOR and 95% CI were calculated as described in the review by Karla [28]. For the remaining studies, because not every study had participants that did not have APBJ or malignancy, the CDOR and 95% CI were too wide for nine studies [3,14,17,28,29,40,74,75,77]. Additionally, the *p*-value was not reported, except by Tomioka [75] (*p* = 0.005 in participants who had high levels of IL-6 and IL-33), who reported the consequence of GB mucosa hyperplasia and the loss of functionality of the greater papilla sphincter. Taken together, more rigorous descriptive studies with more heterogeneous samples are needed to establish any statistically significant correlation.

Moreover, when analyzing the clinical conditions related to the presence of APBJ, the most associated and evidently studied pathophysiological phenomenon is the reflux of pancreatic juice towards the bile duct and the reflux of bile towards the PD, thus appearing as a bidirectional flow and stagnation of the mixture of bile and pancreatic juice. This is because in the APBJ, the sphincter of the major duodenal papilla loses its functionality due to the PBJ being located outside the duodenum in an abnormally long common channel [96]. This anomaly can manifest in various pathological ways that progress to both the biliary tract and pancreas, such as bile duct infections, cysts, pancreatitis, and carcinomas of the biliary tract, GB, and pancreas [4,32,36,38,44,97]. Some studies in this review have shown that APBJ is an important risk factor for the development of GB cancer [98,99] with a prevalence of 14.8% in individuals with APBJ. The prevalence of bile duct cancer is 4.8%, and there is a known association between APBJ and the development of cancer [4,35,38,41,52]. Based on the histological results of this review, it has been shown that the repeated involvement of the biliary epithelium generates genes, activates oncogenes, and inactivates tumor suppressor genes, thereby inducing the hyperplasia–dysplasia–carcinoma sequence [17,100,101]. Thus, it can be deduced that molting induced by a mixture of bile acids and pancreatic juice could play an important role in the formation of GB and bile duct cancers. This is consistent with the information provided in this review and by Funabiki [4], who compared the biochemical composition of bile in patients with APBJ, indicating that they had higher levels of activated pancreatic enzymes, such as amylase, trypsin, lipase, and elastase, compared to patients with normal PBJ, with elevated amylase levels being an altered parameter in patients with APBJ [70,102]. We observed that APBJ variants are a predisposing factor for cancer, similar to the review by Benjamin [43], which stated that the risk of suffering from cancer increases 10% with age, starting from the age of 30. However, our review differs from these by Funabiki [4] and Benjamin [43] because we collected information on the different variants of PBJ and their prevalence. Ohkawa [102] and Ogura [103] have reported that pancreatic enzymes play a carcinogenic role in biliary carcinoma. Similarly, in the context of the association between the APBJ and pancreatitis, dysfunction of the papillary duodenal sphincter leads to the reflux of bile juice towards the PD, causing the premature activation of pancreatic enzymes, which could damage the PD and lead to chronic pancreatitis. Additionally, metaplasia of the PD epithelium can occur, leading to pancreatic carcinoma in patients with chronic pancreatitis [45,47,50,51,60,68]. Although this explanation is the most plausible, it is not the only mechanism for the occurrence of GB pancreatitis and cancer, bile duct cancer, and pancreatic cancer associated with APBJ [56,64].

It is suggested that patients are closely examined when APBJ is suspected based on previous ultrasound findings, such as bile duct dilatation, polypoid GB lesions, or wall thinning [59,102]. The importance of early diagnosis lies in the early detection and more importantly, in the prevention of the development of neoplasia [30,40,60,68]. Several studies have recommended surgical management as initial treatment to prevent complications associated with APBJ. Initially, prophylactic cholecystectomy should be performed when APBJ is diagnosed and a patient presents any of the aforementioned complications or clinical manifestations, to avoid the development of GB cancer [40,60,62,63,68,71,99,100,101,102,103,104,105,106].

### Limitations of the Study

The limitations of this review were the publication and authorship bias of the included studies because studies with different results that were in non-indexed literature in the selected databases may have been excluded. There is also the possibility of not having carried out the most sensitive and specific search regarding the topic to be studied, and finally, there are the individual sessions of the authors for the selection of articles, all of which result in a higher probability of excluding potential cases that are not being reported in the scientific community from countries other than the Asian and North American continents.

## 5. Conclusions

In the present study, we found a relationship between the anatomical variants of APBJ and pathologies such as cancer; the prevalence was 12% for APBJ, and of that that 12%, a 29% prevalence was reported for cancer in subjects with APBJ, which is close to one-third of the population with APBJ. According to the results of this study, the presence of a longer hepatopancreatic duct was specifically correlated with a higher prevalence of malignancy of the bile duct or BV, with statistical significance, all in relation to a retrograde flow of bile and pancreatic juice. Considering the above, we believe that knowing this variant is of utmost importance for surgeons of the hepatic, gastroduodenal, and pancreatic regions, in order to address APBJ in a more effective and timely manner, which will allow a deep understanding to generate better guidelines for the treatment and diagnosis of this type of abnormal condition. Finally, we believe that further studies on this topic could improve knowledge and could explain whether there are more relationships between these anomalous variants and the clinical conditions.

## Figures and Tables

**Table 1:** The sex of the participants in the studies with a sample greater than one.

#	Study	Total Number of Samples	Males, n/%	Females, n/%	Not Reported, n/%
1	Hu et al., 2003 [2]	1876	3/0.15%	7/0.37%	1866/99.46%
2	Itoh et al., 2005 [3]	84	3/33.33%	6/66.66%	0
3	Funabiki et al., 1991 [4]	15	0	0	15/100%
4	Ando et al., 1995 [5]	7	0	0	7/100%
5	Matsumoto et al., 2001 [12]	2018	14/0.69%	49/2.42%	1955/96.87%
6	Kamisawa et al., 2008 [13]	3210	0	0	3210/100%
7	Kochhar et al., 2009 [15]	3827	53/1.38%	48/1.25%	3726/97.36%
8	Komi et al., 1992 [20]	51	17/33.33%	34/66.66%	0
9	Roukounakis et al., 2000 [23]	29	5/17.24%	8/27.58%	16/55.17%
10	Kimura et al., 1985 [29]	211	31/14.69%	65/30.80%	115/54.50%
11	Tanaka et al., 1993 [30]	32	2/6.25%	5/15.62%	25/78.13%
12	Hori et al., 2006 [31]	108	0	0	108/100%
13	Ragot et al., 2017 [32]	263	0	0	263/100%
14	Ono et al., 1982 [33]	22	6/27.27%	9/40.90%	7/31.81%
15	Xia et al., 2015 [34]	86	8/9.3%	19/22.09%	59/68.6%
16	Uetsuji et al., 1996 [35]	47	1/2.12%	6/12.76%	40/85.10%
17	Govil et al., 1998 [36]	18	3/16.66%	6/33.33%	9/50%
18	Tanaka et al., 1998 [37]	38	5/13.15%	9/32.14%	24/63.15%
19	Hanada et al., 1999 [38]	73	10/13.69%	25/34.24%	38/52.05%
20	Yoshida et al., 1999 [39]	650	1/0.15%	13/2.00%	636/97.84%
21	Hosoki et al., 2004 [40]	17	1/5.88%	6/35.29%	10/58.82%
22	Deguchi et al., 2007 [41]	43	2/4.65%	1/2.32%	40/93.02%
23	Alkhatib et al., 2012 [42]	1050	7/0.66%	5/0.47%	1038/98.59%
24	Nakayama et al., 2001 [43]	5	0	5/100%	0
25	Jaunin-Stadler et al., 2002 [44]	2	0	2/100%	0
26	Komi et al., 1977 [45]	570	173/30.35%	397/69.64%	0
27	Young et al., 1992 [46]	9	3/33.33%	6/66.66%	0
28	Kuga et al., 1998 [47]	2	1/50%	1/50%	0
29	Sugiyama et al., 1998 [48]	18	5/27.77%	13/72.22%	0
30	Sugiyama et al., 2000 [49]	31	16/51.61%	15/48.38%	0
31	Takaya et al., 2003 [51]	34	9/26.47%	25/73.52%	0
32	Roukounakis et al., 2006 [52]	58	26/44.82%	32/55.17%	0
33	Guo et al., 2012 [53]	75	24/32%	51/68%	0
34	Liu et al., 2015 [54]	72	37/51.38%	35/48.61%	0
35	Sato et al., 2001 [55]	12	1/8.33%	11/91.66%	0
36	Chapuy et al., 2006 [70]	4	0	4/100%	0
37	Samavedy et al., 1999 [75]	7537	13/0.17%	5/0.06%	7519/99.76%
38	Shimada et al., 1993 [76]	22	6/27.27%	16/61.53%	0

**Table 2:** The prevalence of APBJ in patient cohorts of the studies reviewed with a sample greater than one.

#	Study	Total Number of Samples	Prevalence, n/%
1	Hu et al., 2003 [2]	1876	10/0.53%
2	Itoh et al., 2005 [3]	84	9/10.7%
3	Matsumoto et al., 2001 [12]	2018	202/10.00%
4	Kamisawa et al., 2008 [13]	3210	74/2.30%
5	Kochhar et al., 2009 [15]	3827	101/2.63%
6	Kimura et al., 1985 [29]	211	65/30.81%
7	Hori et al., 2006 [31]	108	12/11.11%
8	Xia et al., 2015 [34]	86	26/30.23%
9	Yoshida et al., 1999 [39]	650	14/2.15%
10	Deguchi et al., 2007 [41]	43	3/6.47%
11	Alkhatib et al., 2012 [42]	1050	12/1.14%
12	Guo et al., 2012 [53]	75	17/22.66%
13	Samavedy et al., 1999 [75]	7537	18/0.23%

**Table 3:** Prevalence of cancer among participants with APBJ.

#	Study	Total Number of Samples	Prevalence, n/%
1	Itoh et al., 2005 [3]	9	3/33.33%
2	Muraki et al., 2017 [14]	64	22/34.4%
3	Kimura et al., 1985 [29]	65	16/24.62%
4	Tanaka et al., 1993 [30]	32	7/21.88%
5	Sai et al., 2006 [71]	11	5/45.5%
6	Tomioka et al., 2019 [72]	32	6/18.75%
7	Kamisawa et al., 2010 [73]	52	19/9.8%
8	Takuma et al., 2012 [74]	36	11/30.56%

**Table 4:** The odds ratio (OR) of cancer development among participants with APBJ in the studies included in the review.

#	Study	Predictor Factor	Cancer Development OR	95% CI	*p*-Value
1	Itoh et al., 2005 [3]	Common channel length (>15 mm on ERCP) Carcinoma and APBJ	0.007	0.16–0.0003	NR
2	Muraki et al., 2017 [14]	Through high amylase levels (>10,000 IU/L) and mucosa histopathology	54	6666.025–0.43	NR
3	Kimura et al., 1985 [29]	Common channel length (>15mm) Carcinoma and APBJ	1.4	3–0.67	NR
4	Tanaka et al., 1993 [30]	Common channel length (>10mm) Carcinoma and APBJ	0.07	2.62–0.0002	NR
5	Sai et al., 2006 [71]	Through high amylase levels (>10.000 IU/L) and common channel length 23.6 mm (SD 9.2)	0.83	6.11–0.11	NR
6	Tomioka et al., 2019 [72]	Through histopathological (fixed in 10% formalin, embedded in paraffin and tinted with H/E) and immunohistochemical methods (for MUC1, MUC2, MUC5AC, MUC6, p53, and SMAD4 with Leica Microsystems) in APBJ	0.012	0.21–0.0007	NR
7	Kamisawa et al., 2010 [73]	Common channel length ≥ 6 mm Gallbladder carcinoma	0.12	6.42–0.002	NR
8	Takuma et al., 2012 [74]	Through histopathological (fixed in 10% formalin, embedded in paraffin and tinted with H/E) and immunohistochemical methods (using antisera for Ki-67) in APBJ	3.21	169.56–0.06	NR

Abbreviations: CI: Confidence interval; SD: standard deviation; ERCP: endoscopic retrograde cholangiopancreatography; and NR: not reported.

## Data Availability

Not applicable.

## Supplementary Materials

The following supporting information can be downloaded at: https://www.mdpi.com/article/10.3390/biomedicines13051039/s1, Table S1: The characteristics of the included studies. Table S2: PRISMA.
